# Examining Shared Pathways for Eating Disorders and Obesity in a Community Sample of Adolescents: The REAL Study

**DOI:** 10.3389/fpsyg.2022.805596

**Published:** 2022-03-31

**Authors:** Nicole Obeid, Martine F. Flament, Annick Buchholz, Katherine A. Henderson, Nick Schubert, Giorgio Tasca, Helen Thai, Gary Goldfield

**Affiliations:** ^1^Children’s Hospital of Eastern Ontario Research Institute, Ottawa, ON, Canada; ^2^School of Psychology, University of Ottawa, Ottawa, ON, Canada; ^3^Department of Psychiatry, University of Ottawa, Ottawa, ON, Canada; ^4^Centre for Healthy Active Living, Children’s Hospital of Eastern Ontario, Ottawa, ON, Canada; ^5^Department of Psychology, Carleton University, Ottawa, ON, Canada; ^6^Anchor Psychological Services, Ottawa, ON, Canada; ^7^Institute of Mental Health Research, Royal Ottawa Mental Health Centre, Ottawa, ON, Canada; ^8^Department of Pediatrics, University of Ottawa, Ottawa, ON, Canada

**Keywords:** eating disorder (ED), obesity, adolescent and youth, risk and protective factors, structural equation model – SEM

## Abstract

Several psychosocial models have been proposed to explain the etiology of eating disorders (EDs) and obesity separately despite research suggesting they should be conceptualized within a shared theoretical framework. The objective of the current study was to test an integrated comprehensive model consisting of a host of common risk and protective factors (socio-environmental, psychological, and behavioral) expected to explain both eating and weight disorders simultaneously in a large school-based sample of adolescents. Data were collected from 3,043 youth (60% female, 14.00 ± 1.61) from 41 schools in the Ottawa region, Canada. Working with interested school staff, validated self-report scales in the form of a questionnaire booklet were administered to participating students to assess several understood risk and protective factors common to both eating disorders and obesity. Anthropometric measurements of weight and height were taken at the end of the questionnaire administration period by trained research staff. Structural equation modeling with cross-validation was used to test the hypothesized model. Findings demonstrated that dysregulated eating was associated with both eating disorder and weight status with diet culture and emotion dysregulation directly associated with some of these disordered eating patterns. It equally pointed to how lifestyle made up of high sedentary behaviors, low vigorous exercise and varied eating patterns contributed to both emotion dysregulation and poor body image which subsequently affected eating issues and weight status simultaneously, signaling the complex interplay of psychosocial factors that underlie these concerns. This study provides evidence for an integrated psychosocial model consisting of socio-environmental, psychological, and behavioral factors may best explain the complex interplay of risk and protective factors influencing eating disorders and obesity. It equally highlights understanding the direct and indirect effects of some of the most salient risk factors involved in eating and weight-related concerns, including the strong effects of diet culture and stressors such as weight-based teasing, providing interventionalists evidence of important risk factors to consider targeting in eating disorder and weight-based prevention efforts.

## Introduction

Adolescence is a developmental period in which individuals experience enormous change in eating behaviors and body weight. These rapid changes are often accompanied by weight and shape preoccupations and some degree of body dissatisfaction, with prevalence in Western countries ranging between 34.1 and 61.8% for adolescent girls and 14.1 and 19.9% for adolescent boys (Bornioli et al., 2021). Body dissatisfaction that derives from changes in eating and weight places adolescents at risk for various health compromising behaviors including inadequate dietary intake, excessive weight gain, and usage of extreme weight control strategies (Jankauskiene et al., 2019). These concerns also place young people at higher risk for added mental health struggles including low mood, anxiety and substance use (Perryman et al., 2018; Vannucci and Ohannessian, 2018; Bornioli et al., 2021). Given the multiple physical, psychological, and social changes that occur during puberty, adolescence represents a time of peak incidence for eating disorders (EDs) (Javaras et al., 2015) and other mental health issues (Boak et al., 2020), and a critical time for the development of lifelong weight concerns (Wang et al., 2008). Investigating shared risk and protective factors for EDs and obesity that could potentially stave off some of these concerns during this vulnerable developmental period could have benefits for these prevalent public health issues facing high numbers of youth.

However, until recently, the etiological and treatment models for EDs and obesity have had different theoretical foundations, leading to polarized clinical and research agendas and conflicting messages for the scientific community and public. Accordingly, prevention efforts for youth in the field of EDs and obesity have sometimes had contraindicated consequences as the ED prevention programs typically targeted dieting, body dissatisfaction and weight talk, whereas interventions aimed at obesity prevention addressed low levels of physical activity and high intakes of food (Neumark-Sztainer, 2012). Attempts to consolidate these efforts across these two public health domains could yield a number of wider scale benefits and impacts across these fields.

In 2003, the Research on Eating and Adolescent Lifestyle study (REAL study; Flament et al., 2019) was initiated to tackle this gap in understanding of the shared risk and protective factors for eating and weight disorders in Canadian youth. This large school-based study endeavored to survey students in grades 7–12, asking about a wide range of factors relevant to eating and weight related issues, in order to validate a complex model of direct and indirect etiological risk and protective pathways for both eating and weight disorders. A comprehensive review of the ED and obesity risk factor literature (conducted in 2003) revealed more than 30 variables from diverse theoretical perspectives emerging as shared risk factors for both EDs (Stice, 2002; Jacobi et al., 2004; Treasure et al., 2010) and obesity (Maffeis, 2000; Berkowitz and Stunkard, 2002; Adair, 2008; McAllister et al., 2009; Sørensen, 2009) all meriting contribution to a model. Potential factors were categorized into four broad domains of risk: biological (including weight status, pubertal status, history of ED or obesity in family member), environmental (including family context, stressors, and societal influences), individual (including self-perception, interpersonal functioning, lifestyle, and emotional regulation/coping), and eating and weight specific behaviors (including restrained eating, dysregulated eating and body image), and were conceptualized to influence each other in direct and indirect ways. Despite this evidence-base, the empirical evidence for contributing factors varied from very strong to very weak; few of the proposed risk factors had been identified as significant in multiple samples; many studies focused on a small number of potential risk factors; and the relative role of different biological, familial, and psychosocial factors in predicting ED onset or obesity was still unclear (Jacobi et al., 2004; McAllister et al., 2009; Sørensen, 2009).

In addition to the risk factor literature that helped to advance knowledge on the underpinnings of eating and weight related disorders, several etiopathological models of EDs that emphasized dual- or multi-factor risk pathways also contributed to the REAL model design. Models, including Garner et al.’s (1983) dual pathway model, Halmi’s (1997) bulimia nervosa (BN) model that stressed the role of dieting and its multiple contributors, Fairburn et al.’s (1999) personal, environmental and dieting vulnerability model, and Thompson et al.’s (2004) tripartite influence model including peers, parents and the media. Further mediation models by Stice et al. linking internalization of the thin ideal to body dissatisfaction and unhealthy dieting behaviors, negative affect and bulimic symptoms (Stice, 2001) and Steiger’s (2004) that placed special emphasis on the exacerbation of genetic vulnerabilities by developmental experiences and current stressors including caloric deprivation and excessive dieting, also contributed to the design of the model. It did so by highlighting the multiple and sometimes mediational pathways that may exist from various risk factors, and the often complex relationships that could emerge amongst a number of risk factors.

With the evidence-base in 2003 limited to etiological theories and studies conducted in EDs or obesity samples separately, two American-based prospective longitudinal studies paved some of the early understanding of the interconnectedness of these factors and their outcomes. Specifically, the Project Eating Among Teens study (Project EAT; Haines et al., 2006), and the Growing Up Today study (GUTS; Field et al., 1999) were both conducted in large community-based samples of United States adolescents. These pivotal studies confirmed a number of shared risk and protective factors for overweight and disordered eating practices among adolescents, although they focused their main outcomes on weight and selected ED behaviors (Project EAT: overweight, binge eating, and extreme weight control; GUTS: overweight, use of laxatives or purging, and binge eating) with much focus during this time being devoted to the “obesity crisis” (Miller et al., 2004). The results of these large-scale longitudinal studies (Project EAT: *n* = 2,516 adolescents followed across 5 years; GUTS: *n* = 16,539 youth followed over 10 years) supported the utility of a joint model to predict overweight and disordered eating outcomes, emphasizing the important role of body image and weight/shape concerns as pivotal factors.

**FIGURE 1:**
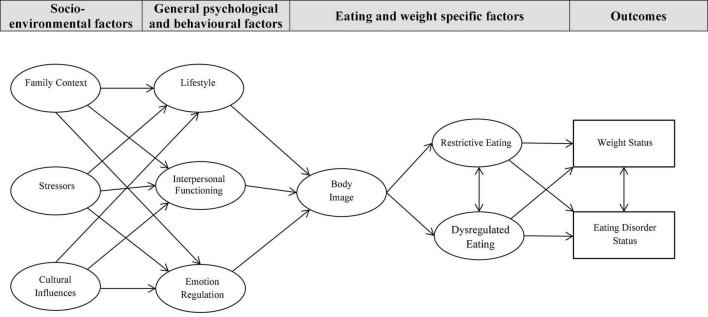
Hypothesized model representing shared pathways to weight and eating disorder status. Family Context: family cohesion, family adaptability, family eating patterns, parents concern with thinness, weight-based teasing from parents; Stressors: negative life events, weight-based teasing from peers, perceived stress; Cultural Influences: internalization of beauty ideals, awareness of beauty ideals, peer concern with thinness; Lifestyle; exercise, sedentary behaviors; Interpersonal Functioning: attachment, non-assertiveness, connection(s) with a supportive person; Emotion Regulation: low mood, anxiety, emotion-oriented coping, inward expression of anger, outward expression of anger; Body Image: appearance esteem, weight esteem, beliefs about appearances; Restrictive Eating: restrained eating; and Dysregulated Eating: emotional eating and external eating.

Together, these literatures and models helped conceptualize an integrated biopsychosocial theoretical framework, the REAL model (see Figure 1). The model incorporated recognized shared risk and protective factors from the literature, direction from existing ED etiological models and learnings from Project EAT and GUTS. This lent to the model being built on the premise that (a) the influence of socio-environmental variables (namely, family context, stressors, cultural influences) on body image in youth would be mediated by general psychological and behavioral factors (i.e., unhealthy lifestyle, poor interpersonal functioning, emotional dysregulation); (b) poor body image would act as a central mediator between the socio-environmental, psychological and behavioral variables and restrained and dysregulated eating; (c) restrained eating and dysregulated eating would share an indirect and direct relationship with ED and weight status, respectively; and (d) socio-environmental factors are understood as some of the propagating factors in these complex relationships.

The objective of the present study was to test this theoretically informed cross-sectional model (outlined in Figure 1), expected to predict eating and weight disorders simultaneously in a large diverse community-based sample of male and female Canadian adolescents. It was anticipated that a unified model including an array of socio-environmental, psychological, and behavioral factors, and specific eating and weight related factors drawn from multiple theories would support pathway prediction for both EDs and obesity in youth in a shared model and that good model fit including direct and indirect pathways would emerge across boys and girls.

## Materials and Methods

### Participants

The study sample consisted of 3,043 male and female students in grades 7 through 12. This age group was selected as it included the two major transition periods of early and late adolescence, times at which youth are at greater risk for emerging eating and/or weight-related issues and accompanying mental health difficulties (Schulenberg et al., 2004). Data were collected from 41 public and three private schools between 2004 and 2010, after all major public-school boards (urban, suburban, and rural), two alternative schools, and three private schools from the capital region of Canada (Ottawa, Ontario, and surrounding area) were approached for participation in a Research study on Eating and Adolescent Lifestyles (*REAL* study; Flament et al., 2019). Within each school that consented to participate, the number of classrooms and students approached were based on school interest and feasibility, generating a convenience sample for the study. Small incentives were provided for student participation. Across schools, the rate of students who provided parental and personal consent and completed the survey was about 45% of those approached. The study was approved by two institutional research ethic boards and the school boards where required.

### Measures

#### Outcome Variables

Weight status was obtained based on measurements taken directly during survey administration while ED status was determined based on self-report.

##### Weight Status

Weight was measured using a UC-321 Digital Weighing Scale (Quick Medical Equipment and Supplies, United States), and recorded in kilograms to the nearest 0.1 kg. Height was measured using a HM200P Portable Stadiometer (Quick Medical Equipment and Supplies, United States), and recorded in centimeters to the nearest 0.1 cm. Students wore indoor clothing and were asked to remove their shoes and anything in their pockets before measurements were taken. Body mass index (BMI; kg/m^2^) was calculated with weight status defined using the International Obesity Task Force (IOTF) criteria based on age and sex-based BMI centile curves (Cole et al., 2000): *(1) thinness grade 2* (BMI < 3rd percentile), *(2) thinness grade 1* (BMI >3rd- < 16th percentile), *(3) normal weight* (BMI >16th- < 85th percentile), *(4) overweight* (BMI >85th- < 95th percentile), and *(5) obesity* (BMI >95th percentile).

##### Eating Disorders Diagnostic Scale (Stice et al., 2000)

This self-report scale was designed to assess DSM-IV criteria for current diagnoses of EDs and includes 22 items adapted from validated clinical interviews. The scoring allows for the indication of ED diagnostic categories in addition to the capture of individuals ED behaviors. The EDDS has been validated in adolescent and adult females and males, and has shown excellent test-retest reliability (kappa = 0.75–0.95), internal consistency (alpha = 0.86–0.91), criterion validity, and convergent validity (Stice et al., 2000, 2004). For this study, the original scoring algorithms were revised to ascertain DSM-5 criteria for AN, BN, BED and Purging Disorder (PD) (Flament et al., 2015a) and the participants were classified into five ED categories: (1) *No disordered eating:* participants who did not endorse any ED related cognitions or behaviors, (2) *ED cognitions*: youth who endorsed ED related cognitions although they denied engaging in any ED behaviors, (3) *ED cognitions and behaviors:* participants who endorsed both ED related cognitions and some behaviors, but did not qualify for a diagnosis of full- or subthreshold ED. This group could be considered a “disordered eating” group, (4) *Subthreshold ED:* participants with subthreshold AN, BN, BED or PD, based on the EDDS algorithms (Stice et al., 2004), and (5) *Full-threshold ED:* Participants with DSM-5 diagnoses of AN, BN, BED, or PD.

##### Predictor Variables

A large number of predictor variables derived from various well-validated self-report measures were included in the conceptual model as guided by previous work. The specific scales or subscales used in the current study as indicator variables are listed in Table 1 according to the latent variables for which they were included. All latent variables were constructed so that higher scores on the indicator variable indicated greater disturbance or psychopathology. Table 1 provides psychometric information of the variables and shows the internal consistency of all measures in the current study sample. For a more detailed description of the measures including psychometric properties, please refer to Flament et al. (2019).

**TABLE 1:** Summary of predictor variables, measures, and their psychometric properties in the study sample.

Latent variables assessment measures	# of items	Response scale	α
**Stressors**			
McKnight Risk Factor Survey IV (MRFS-IV) (Shisslak et al., 1999)			
*Negative life events*	9	yes/no	0.52
*Weight-based teasing from peers*	8	Five-point Likert	0.89
*Weight-based teasing from parents*	2	Five-point Likert	n/a
*Weight-based teasing from other adults*	1	Five-point Likert	n/a

**Diet culture**			
Sociocultural Attitudes Toward Appearance (SATAQ) (Heinberg et al., 1995)			
*Internalization* Items for males	7	Five-point Likert	0.89
Items for females	7	Five-point Likert	0.93
*Awareness* Items for males	4	Five-point Likert	0.81
Items for females	7	Five-point Likert	0.87
Dutch Eating Behavior Questionnaire (DEBQ) (Wardle, 1987)			
*Restrained eating*	10	Five-point Likert	0.92

**Emotional dysregulation**			
Children’s Depression Inventory (CDI) (Kovacs, 1985, 1992)	27	Three-point Likert	0.88
Multidimensional Anxiety Scale for Children (MASC-10) (March et al., 1997)	10	Four-point Likert	0.76
State-Trait Anger Expression Inventory (STAXI) (Spielberger et al., 1985)			
*Anger-in*	8	Four-point Likert	0.77
*Anger-out*	8	Four-point Likert	0.76
Coping Inventory for Stressful Situations (CISS) (Endler and Parker, 1990)			
*Emotion-oriented coping*	7	Five-point Likert	0.84

**Unhealthy lifestyle**			
Godin Leisure-Time Exercise Questionnaire (Godin and Shephard, 1985)	2	frequencies	n/a
Leisure-Time Sedentary Activities Questionnaire (Maras et al., 2015)			0.73
*Week days*	3	Six-point Likert	
*Weekend days*	3	Six-point Likert	
Attitudes and Patterns of Eating (APE) (Quirk-Baillot et al., 2012)			
*Healthy family eating behaviors*	7	Five-point Likert	0.54

**Poor body image**			
Body-Esteem Scale for Adolescents and Adults (BESAA) (Mendelson et al., 2001)			0.92
*Appearance esteem*	10	Five-point Likert	0.88
*Attribution*	5	Five-point Likert	0.80
Beliefs About Appearance Scale (BAAS) (Spangler and Stice, 2001)	6	Five-point Likert	0.93

**Dysregulated eating**			
Dutch Eating Behavior Questionnaire (DEBQ) (Wardle, 1987)			
*External eating*	10	Five-point Likert	0.87
*Emotional eating*	13	Five-point Likert	0.94

### Analytic Plan

Structural equation modeling (SEM) was used to test the hypothesized model. Missing data were assessed before analyses were completed and deemed to be missing at random; they were therefore imputed using the expectation-maximization algorithm (Moon, 1996). All analyses were performed using the Analysis of Moment Structures (AMOS) program version 21 (Arbuckle, 2012). The criteria used to assess model fit were a comparative fit index (CFI) of 0.90 or greater and a root mean square error of approximation estimates (RMSEA) lower than 0.07 (Steiger, 2007). In order to test the pathways found in the model, a bias-corrected bootstrap methodology with 5,000 samples was performed (Mackinnon et al., 2004).

## Results

### Characteristics of the Sample

Participants were 1,789 (58.8%) females and 1,254 (41.2%) males, ranging in age from 11 to 20 years (14.19 ± 1.61). The majority of youth (69%) were of normal weight while 7.7% met criteria for a subthreshold or full-threshold ED based on self-report (see Table 2). Additional clinical characteristics of the sample have been reported elsewhere (Flament et al., 2015a,b).

**TABLE 2:** Demographic and clinical characteristics of sample.

Characteristic	Full sample (*N* = 3,043)
**Age, years**	
Mean ± SD	14.19 ± 1.61
Range	11.08–20.75
**School setting, n (%)**	
Urban	13 (31.0)
Suburban	20 (44.9)
Rural	11 (24.2)
**Parental education, n (%)**	
Both parents have college/higher degree	1528 (50.7)
**Ethnic origin, n (%)**	
North American/European	2208 (74.1)
Asian	266 (8.9)
Middle Eastern	140 (4.7)
Central/South American	96 (3.2)
African	89 (3.0)
Aboriginal	58 (1.9)
Other	100 (3.4)
Bi-ethnic	22 (0.7)
**Body mass index (BMI) categories, n (%)**	
Thinness grade 2	22 (0.7)
Thinness grade 1	155 (5.1)
Normal weight	2096 (69.0)
Overweight	587 (19.3)
Obese	177 (5.8)
**Eating disorder (ED) diagnostic status, n (%)**	
No ED symptoms	1427 (46.9)
ED cognitions only	852 (28.0)
ED cognitions and behaviors	380 (12.5)
Subthreshold ED	259 (8.5)
Full-threshold ED	122 (4.1)

### Measurement Model

The hypothesized measurement model that was tested is depicted in Figure 1. The model consisted of two observed variables (weight status and ED status), and nine latent variables, for a total of 27 indicator variables included in the hypothesized model. A confirmatory factor analysis was conducted in order to test whether the indicator variables could be mapped into their respective latent variables. Results from the initial measurement model revealed a few modifications that needed to be performed in order to achieve an adequate model fit. Seven of the 27 indicator variables were eliminated (Family Cohesion, Family Adaptability, Perceived Stress, Peer Concern with Thinness, Attachment, Silencing the Self, and Supportive Persons), and the final measurement model revealed a good model fit, with a CFI value of 0.901 and a RMSEA value of 0.069 (90% CI:0.067, 0.072).

### Structural Model

A structural model between latent variables was tested for model fit. In order to ensure adequate model fit, the sample was randomly divided in half in order to have two independent study samples available for cross-validation purposes. The tested model consisted of the hypothesized pathways between variables with many indirect and direct pathways indicated. The initial analysis of the structural model revealed a poor model fit to the data. Changes based on non-significant pathways, modification indices and conceptual relevance were conducted until an adequate model fit was found. Through this process, two latent variables were removed (family context and interpersonal functioning) and two latent variables were modified and then combined into one (restrained eating and cultural context became *diet culture*). Using the initial sample, several iterations resulted in a good model fit with a CFI value of 0.903 and a RMSEA value of 0.064 (90% CI:0.061, 0.067). Cross-validation of this model with the second half of the sample revealed a good model fit [CFI value of 0.910 and a RMSEA value of 0.063 (90% CI:0.060, 0.066)].

### Direct Effects

**FIGURE 2:**
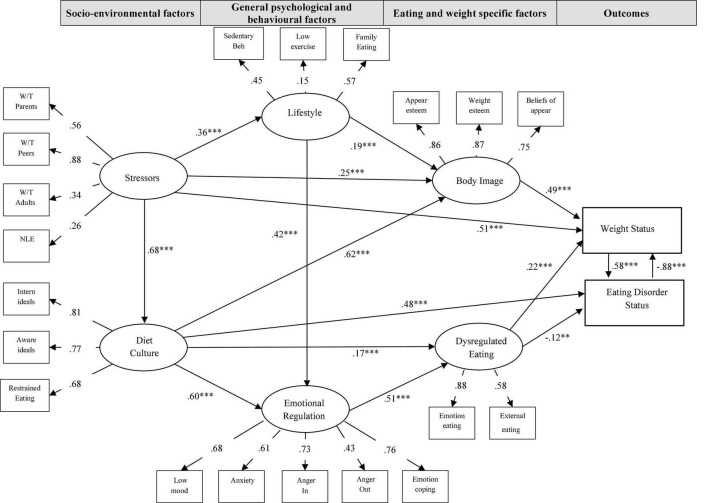
Final structural model of standardized path coefficients. **p < 0.01, ***p < 0.001; W/T Parents: McKnight Risk Factor Survey (MRFS) – Weight-based teasing from parents subscale; W/T Peers: McKnight Risk Factor Survey (MRFS) – Weight-based teasing from peers subscale; W/T Adults: McKnight Risk Factor Survey (MRFS) – Weight-based teasing from other adults subscale; NLE: McKnight Risk Factor Survey (MRFS) – Negative Life Events Subscale; Intern of Ideals: Sociocultural Attitudes Toward Appearance Scale (SATAQ) – Internalization of beauty ideals subscale; Aware of Ideals: Sociocultural Attitudes Toward Appearance Scale (SATAQ) – Awareness of beauty ideals subscale; Restrained Eating: Dutch Eating Behaviour Questionnaire (DEBQ) – Restrained Eating subscale; Sedentary Beh: Sedentary Behaviors Scale; Low exercise: GODIN Exercise Scale; Family Meals: Attitudes and Patterns of Eating (APE) – Family Eating Patterns; Low Mood: Child Depression Inventory (CDI) – Total score; Anxiety: Multidimensional Anxiety Scale for Children (MASC) – Total score; Anger In: State-Trait Anger Expression Inventory (S-TAXI) – Internal Expression of Anger subscale; Anger Out: State-Trait Anger Expression Inventory (S-TAXI) – External Expression of Anger subscale; Emotion Coping: Coping Inventory for Stressful Situations (CISS) – Emotion-focused coping subscale; Appear esteem: Body Esteem Scale for Adolescents and Adults (BESAA) – Appearance esteem subscale; Weight esteem: Body Esteem Scale for Adolescents and Adults (BESAA) – Weight esteem subscale; Beliefs of appear: Beliefs About Appearance Scale (BASS) – Total score; Emotion eating: Dutch Eating Behaviour Questionnaire (DEBQ) – Emotional eating subscale; External eating: Dutch Eating Behaviour Questionnaire (DEBQ) – External Eating subscale.

Review of the final structural model (see Figure 2) revealed five direct effects in total; two direct effects for ED status and three direct effects for weight status with disordered eating having a direct effect to both ED and weight status. Stressors (*B* = 0.51, *p* < 0.001), dysregulated eating (*B* = 0.22, *p* < 0.001) and poor body image (*B* = 0.49, *p* < 0.001) all had significant direct effects on weight status, with higher levels of these resulting in higher BMI status. Higher diet culture engagement also significantly contributed to higher ED status (*B* = 0.48, *p* < 0.001), whereas dysregulated eating (*B* = −0.12, *p* < 0.01) shared a significant inverse direct relationship with ED status, indicating that higher level of these behaviors would predict lower ED category. This unexpected finding is most likely due to a suppression effect that existed between dysregulated eating and ED status given the positive correlation between the variables (0.272, *p* < 0.001). Lastly, a significant reciprocal relationship or feedback loop (Kline, 2011) also existed between weight and ED status.

### Indirect Effects

Several indirect effects were also detected within the structural model. There were four significant indirect effects related to predicting BMI status. Poor body image acted as a mediator between stressors and BMI status (*p* = 0.007), and between diet culture and BMI status (*p* = 0.015), while emotional dysregulation shared an indirect effect with BMI status through dysregulated eating (*p* = 0.008). As stated above, dysregulated eating was inversely related to ED status, which was inversely related to BMI status (*p* = 0.003).

There were four additional indirect effects predicting ED status. Stressors was indirectly related to ED status through diet culture (*p* = 0.012); emotional dysregulation was indirectly related to ED status via dysregulated eating (*p* = 0.004); and both dysregulated eating (*p* = 0.009) and poor body image (*p* = 0.007) were indirectly related to ED status through BMI status. In addition, nine serial mediator pathways to BMI or ED status were found, as illustrated in Figure 2.

## Discussion

To our knowledge, this is the first study to examine a complex model of shared pathways integrating various theoretical facets for EDs and obesity in a large community-based sample of male and female adolescents using structural equation modeling. The hypothesized model was conceptually derived, based on existing theoretical models and previous research findings in both the ED and the obesity field, and was empirically refined and finalized. The model was supported by cross-validation across two randomly split samples from the study population, adding to its generalizability. It provides further understanding of the shared pathways for EDs and obesity during the critical developmental stage of adolescence for both males and females, and brings together the many constructs that have been tested in smaller, more parsimonious models.

Results from the final model (see Figure 2) demonstrated that stressors, diet culture, lifestyle, emotion regulation, body image, and dysregulated eating all have important and interrelated contributions to the risk pathways of eating and weight-related disorders, and remain salient when taking into account all other risk factors in the model simultaneously. The model confirms that at minimum there are 20 respective factors that span across societal, familial, peer, cultural, and individual domains that are all influencing eating and weight related thoughts and behaviors at any given time, providing further evidence of these complex relationships and need for comprehensive interventions designed to tackle many of the risk factors together. The wide-reaching benefits of possible prevention efforts focused on these shared factors could have significant impact for young people at high risk for EDs and weight-based issues.

The confirmed model found that higher levels of stressors (weight-based teasing, negative life events), dysregulated eating and poor body image all directly influenced higher weight status, providing further strength to these known relationships (e.g., Torres and Nowson, 2007; Sominsky and Spencer, 2014; Tomiyama, 2019). The model also revealed that higher diet culture engagement and dysregulated eating related directly to higher ED status, also building on an existing large body of evidence (e.g., Hesse-Biber et al., 2006). Novel are the multiple significant direct associations found that co-exist alongside many other risk factors and their direct effect on ED symptoms and measured weight status. Additionally, diet culture was also indirectly associated with ED and weight status through three addition pathways (i.e., through Emotion Regulation, Dysregulated Eating, Body Image), as was Stressors (i.e., Lifestyle, Body Image, Diet Culture), demonstrating their far-reaching effects on many other related factors, which in turn further contribute to these thoughts and symptoms. These findings are in line with one of the original hypothesis that socio-environmental factors, such as being a recipient of weight-based teasing/body shaming comments and high endorsement of diet culture contribute directly and indirectly to eating and/or weight related issues, seen in some cases to propagate the issues, yet can be seen as modifiable risk factors that can be easy targets of prevention efforts. As such, our final model may be an important finding for prevention and health promotion efforts, wherein media literacy and deconstruction of the billion-dollar dieting industry could be powerful agents of change against disordered eating and weight-related problems.

Building from Project EAT (Haines et al., 2006) and the GUTS’ learnings (Field et al., 1999), findings from this study provide further evidence, in a Canadian youth sample, of shared risk factors for all types of eating disorder symptoms and severity and across youth of all weights and sizes, building on some of this early work. It additionally showed findings consistent with the first hypothesis that socio-environmental factors would be associated with body image and dysregulated eating, although mediated by psychological and behavioral factors.

For example, the pathway from diet culture to emotion dysregulation, to dysregulated eating, to BMI status, and to ED status, in combination with the direct pathway from diet culture to ED status, is both clinically and conceptually relevant. It demonstrates that these socio-environmental factors have significant impacts not only directly, but also indirectly through emotions and other psychological factors, demonstrating their complex etiopathological effects. This finding compliments previous studies that indicate the unique role that emotional dysregulation plays in ED-specific factors that initiate BED and BN (Lavender et al., 2014; Smith et al., 2018). Studying these factors simultaneously within a model allowed for the examination of varied ED risk pathways, wherein different patterns of disordered eating emerged when triggered by sociocultural factors depending on whether they affected an individual either directly or indirectly through emotions. The ability to depict these varying pathways within a shared model in a large sample of youth provides novel findings to the existing literature. Similarly, stressors were indirectly related to ED status through diet culture in one pathway and through BMI status in another pathway. This result emphasizes that stressors alone do not directly relate to ED status, but stressors become an important contributor to ED status in female and male youth through the lens of diet culture and/or through increased BMI status. This could be seen as potentially contributing to a transdiagnostic view of ED status, acknowledging how stress can manifest differently in various individuals (e.g., under or over-eating).

In addition to both body image and dyregulated eating emerging as central mediators in the model, another significant pathway of interest that emerged from this study’s final model was related to the mediated pathway between poor body image and ED status via BMI status. Poor body image was not directly related to ED status when both eating and weight-related disorders are being considered simultaneously, however, poor body image is related to higher BMI status, which in turn is related to higher ED status, suggesting that the onset of EDs is potentially triggered by anticipated weight fluctuations expected during puberty and adolescence. This is an especially critical finding given that weight fluctuations are to be expected during adolescence, puberty and this period of growth. While we know that youth who are overweight report higher levels of body dissatisfaction, chronic dieting, binge eating with loss of control, drive for thinness, and weight control behaviors compared to their normal/healthy weight peers (Goldfield et al., 2010; Russo et al., 2011; Loth et al., 2015; Jebeile et al., 2021), this results suggest that increases in BMI, expected throughout adolescence for all youth, can translate to ED struggles when poor body image is already present, placing a large majority of young people at risk. Taken together, these findings suggest that it may be important to provide high-school age youth accurate information about expected weight fluctuations during adolescence to help normalize these changes and potentially avert eating or weight-related preoccupations driven by these anticipated weight increases.

Lastly, emotional dysregulation also emerged as a key construct within the model. It mediated the effect of diet culture and unhealthy lifestyle, and also impacted directly dysregulated eating. There has been a growing awareness of the importance of emotions (Harrison et al., 2009), mood disorders and emotional avoidance (Dolhanty and Greenberg, 2007) in the obesity (e.g., Korczak et al., 2014) and ED literature (e.g., Fragkos and Frangos, 2013), especially as it relates to etiological patterns for BN and BED (Stice et al., 2017). Having a greater focus on promoting emotional regulation skills in schools and families as it relates to food and bodies (e.g., mindful awareness or mindful eating skills) may enhance prevention of EDs and obesity in youth while promoting practice of these skills in everyday life, which have been shown to have beneficial effects for a host of other mental health areas (e.g., Cloitre et al., 2019).

The confirmation of this complex risk factor model for EDs and obesity across a large sample of youth yields several practical implications. Namely, the findings could be used to inform universal prevention programs for weight-related issues and EDs simultaneously, and should aim to target the multiple yet interconnected constructs of stressors, diet culture, unhealthy lifestyle, emotion dysregulation, dysregulated eating, and poor body image to optimize the prevention of EDs and weight-based concerns among adolescents. The findings also provide further evidence of the complex nature of eating, weight and body image, and provides concrete modeling of the intersectional nature of eating and mental health concerns. This study is also unique in its inclusion of males and females together, an important Contribution To The Fields of obesity and EDs, which have in many instances targeted prevention efforts to gender specific audiences. This allows for some more universal prevention strategies aimed at whole schools or youth populations. Lastly, given the resounding psychosocial effects of weight-based teasing, stressors, internalization of beauty ideals and pervasiveness of restrictive eating practices in today’s society, prevention efforts should gear focus to these highly impactful risk factors.

Despite the study’s strengths, the study also had noted limitations. Namely, the cross-sectional design does not allow for interpretations of causality or temporal sequencing of relationships. Examination of the pathways identified in this study will need to be confirmed using longitudinal designs. Additionally, this study relied on self-report measures to determine ED status rather than clinical interview and utilized a convenience sample. While the large study population was representative of the youth in the region where it was conducted based on Census data (Flament et al., 2015a), results may not generalize to all adolescents. Finally, the model does not include biological, genetic, and potentially important social determinants (e.g., poverty and family burden) of the disorders under study, nor did it include the role of social media given the time of model construction. Future studies should consider inclusion of these factors in the study of integrated models.

The results of this study offer a first attempt at understanding the interconnectedness of these multiple risk factors and their effects on eating and weight status in a shared model, raising awareness around the complexity and multi-factorial nature of contributors to these issues. They also emphasize the importance of continuing to develop integrated models aimed to inform health promotion, policy, prevention, and treatment efforts tackling eating and weight related issues concurrently.

## Data Availability Statement

The datasets presented in this article are not readily available because informed consent from the participants and their legal guardians to share the raw data publicly was never explicitly collected. Requests to access the datasets should be directed to NO, nobeid@cheo.on.ca.

## Ethics Statement

The studies involving human participants were reviewed and approved by the Royal Ottawa Mental Health Centre and Children’s Hospital of Eastern Ontario Research Ethics Board and the Ottawa-Carleton District School Board Research Advisory Committee. Written informed consent to participate in this study was provided by both the participant and their parent/legal guardian.

## Author Contributions

NO, MF, AB, KH, and GG conceived and designed the work that led to the submission and acquired data. NO and GT played an important role in analyzing and interpreting the results. NO, MF, NS, and HT drafted and revised the manuscript. All authors revised and approved the final version and agreed to be accountable for all aspects of the work.

## Conflict of Interest

The authors declare that the research was conducted in the absence of any commercial or financial relationships that could be construed as a potential conflict of interest.

## Publisher’s Note

All claims expressed in this article are solely those of the authors and do not necessarily represent those of their affiliated organizations, or those of the publisher, the editors and the reviewers. Any product that may be evaluated in this article, or claim that may be made by its manufacturer, is not guaranteed or endorsed by the publisher.
